# Removal of an Upper Third Molar from the Maxillary Sinus

**DOI:** 10.1155/2015/517149

**Published:** 2015-02-01

**Authors:** Klinger de Souza Amorim, Vanessa Tavares da Silva, Rafael Soares da Cunha, Maria Luisa Silveira Souto, Carla Rocha São Mateus, Liane Maciel de Almeida Souza

**Affiliations:** Dentistry Department, UFS Universidade Federal de Sergipe, Avenida Doutor José Thomas D'Ávila Nabuco, 700 Bloco 13, Apartamento 103, Bairro Farolândia, 49030-270 Aracaju, SE, Brazil

## Abstract

The maxillary sinus or antrum is the largest of the paranasal sinuses. It is located in the maxillary bone and has a proximity to the apexes of upper molars and premolars, which allows it to form a direct link between the sinus and the oral cavity. Dislocation of a foreign body or tooth to the interior of a paranasal sinus is a situation that can occur as a result of car accidents, firearm attacks, or iatrogenic in surgical procedures. Therefore, it is necessary to know how to treat this kind of situation. This study's objective is to report the case of a 23-year-old female patient, leucoderma, who sought treatment from the Surgical Unit at the Dental Faculty of the Federal University of Sergipe. She had a history of pain and edema in the right side of the genian region and two failed attempts at removing dental unit (DU) 18. The extraoral clinical exam revealed intense edema of the left hemiface with signs of infection, excoriation of the labial commissure, hematoma, a body temperature of 39°C, and a limited ability to open her mouth. The patient was medicated and treated surgically. The tooth was removed from the maxillary sinus with caution, as should have been done initially.

## 1. Introduction

The maxillary sinus, or antrum, is the widest of the paranasal cavities, occupying the entire maxillary body. It is described as a triangular pyramid whose base is the lateral nasal wall and whose apex faces the zygomatic process of the maxillary bone [1, 2]. It has a large volume, capillary fragility, and proximity to the apexes of some of the upper teeth, namely, the molars and premolars, which allows it to form a direct connection between the sinus and the oral cavity.

Although foreign bodies within the paranasal sinuses are observed rarely, objects can become lodged in the paranasal sinuses as a result of car accidents, firearm attacks, psychiatric disorders, or iatrogenic in surgical procedures. The upper third molar can become dislocated within the maxillary sinus cavity during dental surgery; this rare circumstance accounts for 0.6–3.8% of iatrogenic cases of foreign body entrapment in paranasal sinuses [3]. When such a dental dislocation does occur, it can usually be attributed to inadequate use of extractors in the context of an atypical anatomical relationship between the tooth and the maxillary sinus.

Imaging exams are necessary to help in the diagnostic process. The most commonly used method is orthopantomography, but Water's method, side profile, and computed tomography are also used [4]. When dislocation of a tooth to the maxillary sinus is diagnosed, surgical planning to remove it is needed.

This report describes a clinical case of a patient whose third molar was iatrogenically dislocated to the interior of the maxillary sinus. So the clinical conduct and surgery used to resolve this case are presented at the report.

## 2. Case Report

A 23-year-old female patient, leucoderma, sought treatment from the Surgery Unit at the Dental Faculty of the Federal University of Sergipe. She had a history of pain and edema in the right side of the genian region after two failed attempts to remove the dental unit (DU) 18. She brought in a panoramic X-ray taken prior to those two surgeries.

**Figure 1:**
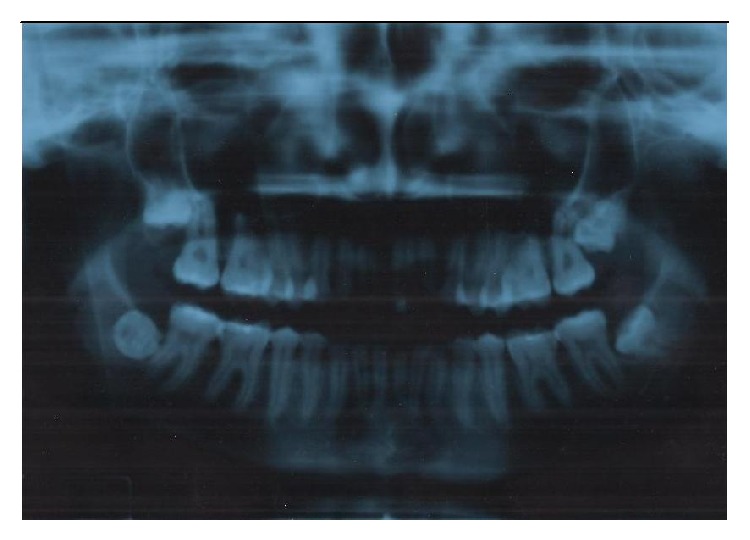
X-ray before any intervention.

During the extraoral clinical exam, intense edema of the left hemiface with signs of infection, excoriation of the labial commissure, hematoma, and a limited ability to open the mouth were observed. The case was characterized as level 1 seriousness [5]. The patient had a body temperature of 39°C. On the preoperative X-ray exam, the presence of dental inclusion for DUs 18, 28, 38, and 48 was observed. DU 18 had vertical impaction and intimacy with the maxillary sinus, and DU 28 had mesioangular impaction. DU 38 had horizontal impaction (class 2 branch and class B depth), and DU 48 was totally vestibularized with horizontal impaction (class 2 branch and class B depth) [6] (Figure 1).

As an initial measure, antibiotics (1000 mg/5 mL amoxicillin + 250 mg/5 mL sulbactam every 12 hours over 7 days) and analgesic (500 mg paracetamol + 7.5 mg codeine phosphate every 6 hours) were prescribed. Supplement support consisted of 01 g of vitamin C once a day and 300 mg of B complex once a day. The patient was directed to place wet, lukewarm compresses on the sore area for 5 days and to remain hydrated by consuming 250 mL of liquid every 3 hours.

**Figure 2:**
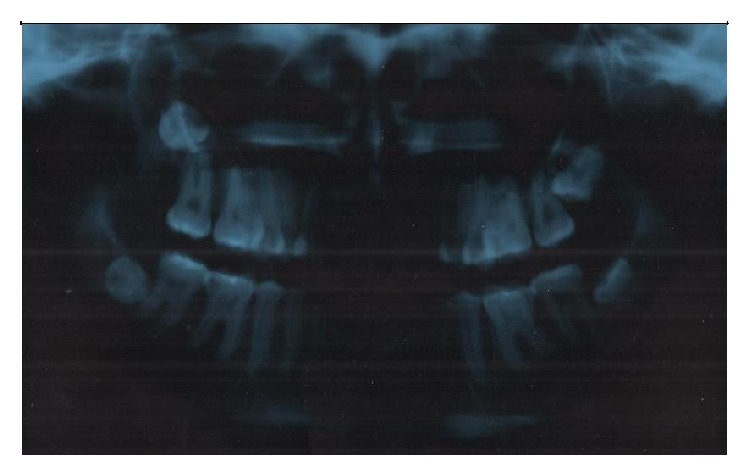
X-ray with the DU 18 into the maxillary sinus.

A new panoramic X-rays was requested to clarify the diagnosis. One week later, the patient has overcome her infection with new X-rays that showed the presence of tooth 18 in the interior of the maxillary sinus (Figure 2). Because of the trauma she had endured from her previous surgeries, it was administered Corah's dental anxiety scale [7], which showed that she was moderately anxious.

**Figure 3:**
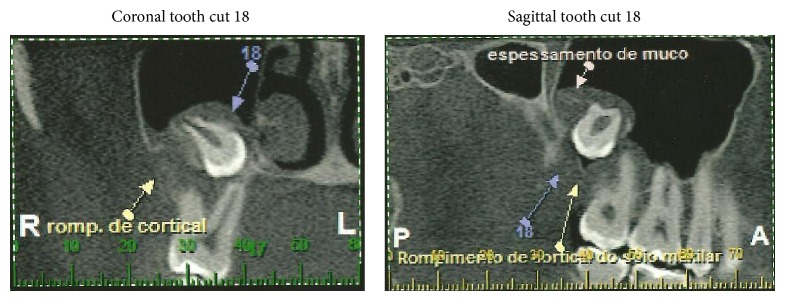
Computed tomography examination of the maxilla by volumetric acquisition to better locate DU 18.

The patient was sent to receive a computed tomography examination of the maxilla by volumetric acquisition to better locate DU 18 (Figure 3). After tomography, the surgical planning was conducted. Three surgical procedures were planned to remove DUs 28, 38, and 48, and the displaced DU 18 from the maxillary sinus. One-week interprocedural interval was applied between the interventions.

For the first two surgeries, 500 mg of the phytotherapeutic mulungu 30 minutes before surgery was given to the patient as an anxiolytic measure. For the third surgery for removal of DU 18 from the maxillary sinus, 15 mg of midazolam was given 30 minutes before surgery to reduce her anxiety. As prophylactic antibiotic therapy, 2 grams amoxicillin was given to her 1 hour before surgery [8]. For all three operations, a protocol using dexamethasone (8 mg, intramuscularly) was chosen 30 minutes before the procedure as a prophylactic against postoperative edema [9]. The patient's arterial pressure (AP), heart rate (HR), and oxygen saturation (O_2_ Sat) were monitored throughout all three surgeries and they were maintained without changes.

The anesthesia for the operation on DUs 38 and 48 was achieved with 2% lidocaine + 1 : 100,000 epinephrine (1.8 mL) in a Vazirani-Akinosi block and 4% articaine with 1 : 100,000 epinephrine (0.9 mL) in an oral nerve block. The drugs were injected as preconized at current literature [10] and the surgical technique for DUs 38 and 48 followed the recommended protocol [11].

The anesthesia for the operation to remove DU 28 was achieved by application of an upper posterior alveolar nerve block with 4% articaine + 1 : 100,000 epinephrine. It was taken 10 minutes after applying the alveolar nerve anesthetic before proceeding with the surgery to avoid the need to block the greater palatine nerve [12]. The surgical technique followed the preconized protocol [11]. 750 mg of paracetamol every 6 hours for 24 h was prescribed as a postsurgical analgesic [13]. For the third operation, to remove DU 18, a blockage of the upper posterior and middle alveolar nerve with 4% articaine + 1 : 000,000 epinephrine, waiting 10 minutes for palatal diffusion, was made [12]. The tooth was removed from the maxillary sinus using the Caldwell Luc operation, which was originally described in the late 1800s as an approach to the maxillary sinus accessing it through the labiogingival sulcus to canine fossa incision. By this technique, there is bone resection of the sinus vestibular wall, which is open and provides access to the removal of the foreign body from the maxillary sinus, and then the sinus must be irrigated and the suture must be made, relaxing at health bone 6, 10. Postoperatively, another 8 mg dose of dexamethasone to take in the evening and paracetamol (750 mg every 6 hours for 24 hours) were prescribed.

The following postoperative instructions were explained to the patient for each of the surgeries she received.

1. Avoid exposure to sun, hot and hard food, and physical exertion, at least until removal of the stiches.
2. Consume liquid or soft and cold food only for at least 48 hours (milk, juice, etc.).
3. Rest and sleep with your head elevated (remain seated when resting and place pillows under your head at bedtime), and avoid lowering it.
4. Resume normal brushing of the teeth and tongue, but avoid the operated area.
5. Rinse gently 3 times a day with an oral antiseptic, starting 24 hours after the surgery.
6. Use ice compresses externally (on the face) during the first 24 hours for periods of 4 minutes followed by rest for 20 minutes.
7. Spread Vaseline or lip protector on the lips to keep them lubricated and prevent chapping.
8. If you suffer from a high fever, edema, difficulty opening your mouth for more than 3 days, persistent pain, or excessive bleeding, get in touch with us immediately.
9. Adhere rigorously to the prescribed medication schedule.

**Figure 4:**
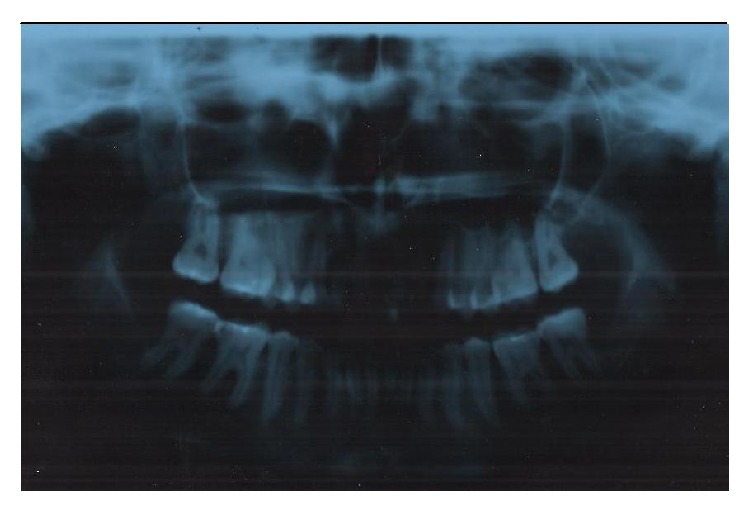
X-ray for postoperative evaluation.

After the final surgery, a new panoramic X-ray for postoperative evaluation was obtained, and it showed no evidence of any complications (Figure 4).

## 3. Discussion

Because of their proximity to the upper teeth, the maxillary sinuses are the most important paranasal sinuses in dentistry [14, 15]. The close relationship between these anatomical structures requires a surgeon who is sufficiently cautious and sensitive to deal adequately with the case. Often, the distance between the root apex and the sinus mucosa is showed to be reduced to millimeters, making the transposition of a dental element to the maxillary sinus possible, mostly the upper third molar [6, 16, 17], as can be seen in this report. According to some authors the present iatrogenic case can be caused by excessive apical force during the use of extractors and incorrect surgical techniques [18, 19]. However, in these circumstances, the professionals need to be prudent and cautious in their handling of the case to reduce the trauma caused by the accident and to attain the desired result [16].

The panoramic X-ray was used for this diagnostic, and it is the most common imaging approach used to confirm the location of elements dislocated to the maxillary sinus, although it can cause a distortion of around 25% [20]. Nevertheless, computed tomography offers the clearest view and a three-dimensional view, which makes it indispensable for the evaluation and proper handling of cases such as the one presented here [21, 22]. In the present case, panoramic X-ray and computed tomography were used to determine with precision the location of the translocated tooth. However, in some cases, the procedure can be completed with only panoramic X-rays [16, 23].

In cases of accidental dislocation of fragments to the interior of the maxillary sinus, some authors agree that the most acceptable treatment is removal to prevent future infection [17, 24–26]. However, infection is not a certain outcome since sinuses have been observed to be healthy despite the inclusion of foreign material [27]. The ideal circumstance is that any dislocated foreign body be taken out during the same surgical procedure in which it was dislodged, if possible. However, the sinus can remain asymptomatic for several months before an acute infection develops. The patient in the present report showed classic signs of an infection caused by dental material inside the right maxillary sinus. Peterson et al. recommend that a tooth dislocated to the maxillary sinus be removed after a period of 4 to 6 weeks, since there is fibrosis during the initial healing period that can stabilize the tooth, making its positioning firmer [6].

In the present case, the Caldwell-Luc operating technique was used. The main advantages of this technique in this case were good visualization of the operative field (which facilitates better access to the sinus), prior experience with it in the surgeon's routine, and the absence of serious complications. Despite the fact that there are references in the literature about facial asymmetry, nervous lesion, dental pulp devitalization, and oroantral fistulas being complications associated with the Caldwell-Luc procedure [28], there is a scientific consensus that such morbidities are related not only to the technique used but also to the surgeon's experience [29]. Another well-known technique is the transalveolar procedure, but it is only indicated when the already existing opening is larger than the foreign body to be removed [19]. Normally, it is used only as the first and immediate attempt to recover root remains [18, 30]. The advent of endoscopy has also helped with the process of removing small foreign bodies from paranasal cavities [6, 31]. It allows sufficient visualization of the surgical field, has low morbidity, and is easily accepted by patients. However, a lack of specialized manpower and the lack of logistical resources available in most public and private services have made routine use of endoscopy impractical [16].

The results of the patient's Corah scale anxiety test indicated that she was moderately anxious [7]. Moreover, she said that she was very anxious about the surgery to remove the tooth from the maxillary sinus, since previous surgeries on that D.U. had not been successful. Therefore, a benzodiazepine anxiolytic (15 mg midazolam) was given before that final operation. For the other extractions, the patient was calmer. Therefore, for those earlier procedures, a phytotherapeutic anxiolytic (two 500 mg capsules of* Erythrina mulungu*) was prescribed because it does not affect motor coordination [32].

Iatrogenic can occur in various fields of dentistry, even during relatively noninvasive treatments. Therefore, all patients undergoing dental procedures should be told about the risks and possibilities of complications. In this case, removing the tooth dislocated to the maxillary sinus required a second surgical period. Nonetheless, the professional on this case was able to proceed with X-ray planning, surgery, and appropriate medication. The best way to avoid dental dislocation to the maxillary sinuses is through careful preoperative evaluation. Before every surgical procedure, dental teams should request the appropriate complementary exams and evaluate whether the professional has the ability to perform the procedure that needs to be done in a sufficiently cautious way. They also need to make sure that any complications can be resolved, if possible, thereby leaving the patient free from disorders, such as that described in this case report.
